# Classification of laryngeal diseases including laryngeal cancer, benign mucosal disease, and vocal cord paralysis by artificial intelligence using voice analysis

**DOI:** 10.1038/s41598-024-58817-x

**Published:** 2024-04-23

**Authors:** Hyun-Bum Kim, Jaemin Song, Seho Park, Yong Oh Lee

**Affiliations:** 1 Department of Otolaryngology-Head and Neck Surgery, The Catholic University of Korea, Seoul, South Korea; 2 Department of Industrial and Data Engineering, Hongik University, Seoul, South Korea

**Keywords:** Voice, Voice change, Laryngeal neoplasm, Vocal paralysis, Artificial intelligence, Health care, Risk factors

## Abstract

Voice change is often the first sign of laryngeal cancer, leading to diagnosis through hospital laryngoscopy. Screening for laryngeal cancer solely based on voice could enhance early detection. However, identifying voice indicators specific to laryngeal cancer is challenging, especially when differentiating it from other laryngeal ailments. This study presents an artificial intelligence model designed to distinguish between healthy voices, laryngeal cancer voices, and those of the other laryngeal conditions. We gathered voice samples of individuals with laryngeal cancer, vocal cord paralysis, benign mucosal diseases, and healthy participants. Comprehensive testing was conducted to determine the best mel-frequency cepstral coefficient conversion and machine learning techniques, with results analyzed in-depth. In our tests, laryngeal diseases distinguishing from healthy voices achieved an accuracy of 0.85–0.97. However, when multiclass classification, accuracy ranged from 0.75 to 0.83. These findings highlight the challenges of artificial intelligence-driven voice-based diagnosis due to overlaps with benign conditions but also underscore its potential.

## Introduction

The occurrence of laryngeal cancer has decreased alongside a decline in smoking populations, but it still affects over 10,000 people annually in the United States^1,2^ and more than 1000 people in South Korea^3^. The laryngoscope, which must be used by a specialist in a well-equipped hospital, still remains the most critical diagnostic. The most common, and sometimes the sole symptom of laryngeal cancer is voice change, however, the voice is not yet widely used to diagnose the laryngeal cancer. First of all, studying voice is very challenging due to the uniqueness of each individual and the wide diversity in the normal range of voice. Speech production involves airflow from the lungs passing through the larynx, enveloping the vocal cords, and reaching the oral cavity. During this process, a number of factors affect voice quality, including lung capacity, vocal cord size, vocal cord mass, and vocal cord length, which vary depending on variables such as gender, age, hormonal fluctuations, and other determinants^4,5^. Furthermore, the voice can change due to factors such as vocalization technique, personal habits, and environmental conditions, and it also changes when expressing emotions^6^. Furthermore, from a disease perspective, voice changes occur not only in laryngeal cancer, but also in benign mucosal diseases, such as vocal polyp, vocal nodule, and reinke’s edema. Despite the plethora of research endeavors concerning voice as a non-invasive human biomarker^7–9^, discernible distinctions between laryngeal cancer and other laryngeal diseases exist, yet an accurate biomarker has not been identified.

Despite of the limitation of voice-based laryngeal disease diagnosis, voice-based laryngeal cancer diagnostic technology is important and becoming a reality for the following reasons. Through the experience of COVID-19, with an increasing number of patients hesitating to visit hospitals, the necessity of remote medical consultant has been heightened, extending beyond telemedicine. In this process, study on voice using artificial intelligence (AI) has become essential, and this is expanding to encompass not only voice changes caused by laryngeal disease, but also arising from psychological factors such as depression^10^, parkinson’s disease^11^, and more.

There have been some attempts to diagnose laryngeal cancer by AI. Kim et al.^12^ distinguished laryngeal cancer and healthy controls with 0.85 accuracy in one-dimensional convolutional neural network (1D-CNN) using voice data. Kwon et al.^13^ acquired 0.95 of accuracy in the fusion of the laryngeal image and voice data by ensemble learning of two CNN models for the same task, but the accuracy was limited to 0.71, when classifying with only voice data. These voice-based AI laryngeal cancer diagnosis models have the limitation of simply classifying laryngeal cancer and healthy condition.

In this study, we hypothesized that AI could distinguish laryngeal cancer from not only healthy voice but also several laryngeal pathologic voices. Our contributions are following. (1) This study represents the first attempt to develop a classification model exclusively utilizing negative samples from a dataset encompassing various laryngeal disorders, with a specific focus on laryngeal cancer. While previous attempts have been made to classify various laryngeal disorders using only voice data, it is worth noting that laryngeal cancer was not present in this dataset^14,15^. Otherwise, studies have primarily utilized image data to classify various laryngeal disorders, including laryngeal cancer^16–18^. (2) We analyze the differences in signal characteristics between laryngeal cancer, vocal cord paralysis, benign mucosal diseases, and healthy data and provide comprehensive experimental results for all combinations of the four classes for laryngeal cancer diagnosis based on these characteristics.

## Methods

### Study subjects

A retrospective review of medical records and voice samples was performed at a single university center from January 2015 to December 2022. We identified patients with voice change over three weeks who underwent voice assessment during their initial visit to our clinic. Only preoperative or pre-procedure results were collected. Patients under 19 years of age, pregnant women, and those who had previously undergone surgery or radiotherapy on their vocal cords were excluded. Furthermore, all women were excluded because voice is affected by sex significantly and the appearance of laryngeal disease varies depending on sex. All healthy voice samples were collected during voice assessment prior to general anesthesia surgery for another site such as thyroid, and lung. All healthy subjects reported having no voice-related issues, and were confirmed through the laryngoscope performed by two otorhinolaryngologists that there was no lesion on vocal cords. All abnormal voices were classified by the clinical purposes considering pathologic results: laryngeal cancer, benign mucosal disease, and vocal cord paralysis. Laryngeal cancer includes all malignant changes in the larynx. Benign mucosal disease includes several diseases such as vocal polyp, vocal nodule, granuloma, and reinke’s edema Vocal cord paralysis includes decreased mobility of uni or bilateral vocal cords regardless of complete closure. The treatment after diagnosis differs significantly among these diseases: Laryngeal cancer typically necessitates radiotherapy or surgery, while benign mucosal disease is usually managed with voice therapy or microlaryngeal surgery

### Data collection

The voice samples were recorded with a Kay Computer Speech Lab (CSL) (Model 5121; KayPentax, Lincoln Park, NJ, USA) supported by a personal computer, including a Shure-Prolog SM48 microphone with Shure digital amplifier, located at a distance of 10–15 cm from the mouth and an angle of 90 degree. Although recording was not done in soundproof room, background noise was controlled below 45 dB HL. Analysis of a voice sample, directly recorded using digital technology and with a sampling frequency of 50,000 Hz, was carried out using MDVP 5121 (version 3.3.0). Participants phonated vowel sound/a $:$/for over 4 seconds at a comfortable level of loudness (about 55–65 dB HL). And we also measured aerodynamic parameters using phonatory aerodynamic system (PAS) (Model 6600; KayPentax, Lincoln Park, NJ, USA). An intraoral tube with an inner diameter of 1.651 mm and an outer diameter of 2.413 mm was inserted into the adult mask and then attached to the face so that the mask completely covered the subject’s nose and mouth. Subsequently, /pa/ was produced a total of 5 times, once per second, at a comfortable voice height and volume similar to normal conversation. The average value of the middle 3 utterances was analyzed.

The study was approved by Yeouido St. Mary Hospital of the Catholic University of Korea institutional review board (IRB) (Development of Artificial Intelligence Platform for an Early Diagnosis of Laryngeal Cancer by Combining Speech and Image, XC23RIDI0042, approved July 25, 2023). This is a retrospective study that analyzed the participants’ past clinical history and recording files. There were no disadvantages to the participants or infringement on their personal information. Although informed consent was not directly obtained from participants due to the retrospective nature of the study, the IRB provided a waiver for informed consent. All procedures were in accordance with IRB ethical standards and the Helsinki Declaration of 1975.

### Feature extraction

Mel-Frequency Cepstral Coefficients (MFCCs) were utilized as the chosen technique for obtaining transformed representations of the speech data. To enhance the dataset, the full speech signals were partitioned into discrete intervals of 0.5 s. Subsequently, each individual interval was subjected to the conversion process.

**Figure 1:**
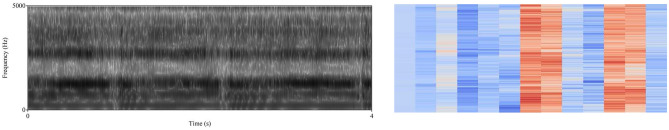
Example of converting voice signal (laryngeal cancer) to MFCC.

During the MFCC transformation process, the segmented speech intervals were analyzed using a sliding window approach. The window duration was established at 0.02 s, with an overlap of 0.01 s, and the conversion was achieved by advancing the window by 0.01 s incrementally. This method led to the extraction of a total of thirteen MFCC coefficients from the given speech data. Figure 1 is an example of MFCC conversion.

### Machine learning and deep learning algorithms

Three distinct algorithms were employed in this study: Support Vector Machine (SVM), LightGBM, and Artificial Neural Network (ANN).

SVM^19^, a renowned classification algorithm, was applied to categorize data by identifying the optimal hyperplane for segregation in high-dimensional or infinite-dimensional spaces. In the context of our experiments, we addressed data imbalance by configuring the ‘class$\_$weight’ parameter as ‘balanced’. This adjustment aimed to alleviate the impact of data distribution disparities, potentially bolstering accuracy through the imposition of penalties on prediction errors committed within underrepresented classes. The input for SVM comprised MFCC image data.

LightGBM^20^, another algorithm employed, features a unique leaf-wise tree splitting approach. This entails prioritizing the division of nodes with the greatest prediction error among the leaf nodes. Notably, for the augmentation of the laryngeal cancer voice data-a category with the least representation within the experiment-the ‘class_weight’ for laryngeal cancer voice was set to 5, while the remaining categories were assigned a weight of 1. The input for LightGBM consisted of the vector values derived from the computation of MFCC coefficients.

Lastly, the Artificial Neural Network (ANN)^21^ architecture was composed of multiple fully connected layers. Within the experiment, the configuration for hidden layer sizes was set as (32,8).

The use of ResNet50^22^, denoted as a CNN algorithm, was integral to the execution of this investigation. The Resnet50 model underwent preliminary pre-training on the image-net dataset. Following the application of the Resnet50 model, a pivotal step involved the incorporation of a Global Average Pooling layer. This architectural element was adeptly employed to orchestrate the conversion of the initial 3D feature map into a singular 1D vector representation.

A strategic emphasis on mitigating overfitting was evident through the strategic integration of a dropout layer, the operational configuration of which featured a dropout ratio set at 0.5. This strategic decision materialized in the execution of two distinct dropout procedures, subsequently applied to the ensuing dense layer.

The framework of the model entailed two fully connected (Dense) layers, each distinguished by a composition of 64 and 16 nodes, respectively. This architectural structure was fortified by the prudent incorporation of batch normalization and the ReLu activation function. Additionally, meticulous attention was directed towards configuring the terminal layer’s node arrangement, ens uring alignment with the discrete count of classes germane to the classification conundrum.

### Model performance evaluatation

In pursuit of effective data validation, the dataset was bifurcated into two distinct subsets: training data and testing data. Notably, the validation dataset was primarily leveraged to validate the efficacy of the training procedure. Consequently, its influence on weight updates remained marginal, and the computation of performance metrics was exclusively limited to the test dataset.

To address the inherent limitation in data availability, a 5-fold cross-validation approach was adopted. This strategy involved partitioning the complete dataset into five discrete segments. Subsequently, one-fifth of the data was allocated for validation purposes, leaving the remaining four-fifths as training data. This iterative process was executed five times, yielding five distinct sets of performance metrics. The composite performance metric was then derived from the average of these computed outcomes.

A comprehensive suite of evaluation metrics was employed to assess the models’ performance. These metrics encompassed accuracy, precision, recall, and f1-score.

## Results

We enrolled a total of 363 patients and analyzed their /a/ vocal samples. The average age of all patients was 52 years, and a significant difference was observed between the laryngeal cancer group and the other diseases. The rate of heavy smokers was also significantly higher in the laryngeal cancer group. Interestingly, there was no significant difference in alcohol intake among all the groups. All 30 cases of laryngeal cancer were confirmed pathologically as squamous cell carcinoma. 23 had lesions located on a unilateral vocal cord. 17 patients, including 7 patients who had bilateral vocal cord lesions were confirmed by the stroboscopy to affect the anterior commissure. 25 cases were limited to glottis and the others were diagnosed as transglottic cancer. 26 cases were classified as early stage according to the eighth edition of the AJCC TNM classification. Among 97 cases of vocal cord paralysis, only one case was diagnosed as bilateral vocal cord paralysis, who underwent the tracheostomy later. 27 patients had lesions located on bilateral vocal cords, including reactive nodules and reinke‘s edema among 81 benign mucosal diseases. 18 patients were confirmed by the stroboscopy to affect the anterior commissure. The detail information about participants is given in Table 1.

**Table 1:** Clinical characteristics of the 363 male participants.

Characteristics		Classification$\ddagger$				p-value
		N ($\%$)* or Mean ± SD				
		Laryngeal Cancer	Vocal cord paralysis	Benign mucosal disease	Healthy	
		(N = 30)	(N = 97)	(N = 81)	(N = 155)	
Age (years)		66 ± 11	55 ± 18	54 ± 14	46 ± 13	<0.01
Smoking ($\%$)	< 30 pyrs**	6 (20)	75 (77)	68 (84)	144 (92)	<0.01
	$\ge$ 30 pyrs	24 (80)	22 (23)	13 (16)	11 (7)	
Alcohol intake	< 7 glasses/week	22 (73)	85 (87)	63 (78)	131 (85)	0.2
	$\ge$ 7 glasses/week	8 (27)	12 (13)	18 (22)	24 (15)	
Lesion	Unilateral	23 (77)	96 (99)	54 (67)		
	Bilateral	7 (23)	1 (1)	27(33)		
Anterior commissure involvement	Yes	17 (57)		18 (22)		
	No	13 (43)		63 (78)		
Malignancy lesion	Glottis	25 (83)				
	Transglottis	5 (17)				
Malignancy T stage	1	24 (80)				
	2	2 (7)				
	3	3 (10)				
	4	1 (3)				

*Percentage of the each of the groups.

**pyrs, an abbreviation for pack years, denotes the consumption of one pack of cigarettes daily over a period of 1 year.

$\ddagger$8th Edition of the AJCC TNM classification (2016).

In our study, we utilized data from 363 participants, comprising 30, 97, 81, and 155 patients individuals each diagnosed with laryngeal cancer, vocal cord paralysis, benign mucosal diseases, and those without any conditions.

The /a/ vocal samples were transformed into MFCC. We subsequently trained these using various machine learning models, including Support Vector Machine (SVM), Light Gradient Boosting Machine (LightGBM), Artificial Neural Networks (ANN), and Convolutional Neural Networks (CNN).

### Voice signal analysis

There are two ways to measure similarity of two signals: distance measurement method and the similarity function^23^. Commonly used distance measure methods are Euclidean distance, Minkowsky distance. Similarity function is divided into binary vector similarity function method and the general vector similarity function. Commonly used general vector similarity function is cosine and correlation coefficient.

Let vector ${{\textbf {x}}} =(x_1,x_2 \ldots x_n),{{\textbf {y}}}=(y_1,y_2 \ldots y_n)$, the similarity measurements between x and y are defined as follows.

- Minkowski distance $$d({\textbf {x}},{\textbf {y}})= \left\{ \sum _{j=1}^{n}\vert x_{j}-y_{j}\vert ^{p}\right\} ^{1/p}$$
- Euclidean distance $$d({\textbf {x}}, {\textbf {y}})=\left[ (x-y)(x-y)^{\prime }\right] ^{1/2}$$
- Cosine distance $$d({\textbf {x}},{\textbf {y}})=1-xy^{\prime }/(x^{\prime }x)^{1/2}(y^{\prime }y)^{1/2}$$
- Correlation distance $$d(x,y)=1-r(x,y)$$

where $$r(x,y)={\textrm{cov}(x,y)\over \sqrt{\textrm{cov}(x,x)\cdot \textrm{cov}(y,y)}}$$ and $$\textrm{cov}(x,y)={1\over {}{_{\displaystyle n{-}1}}}\sum \limits _{i=1}^{n}\left( x_{i}-\overline{x}\right) \left( y_{i}-\overline{y}\right)$$

The four metrics for the similarity of laryngeal disease data to healthy voice data are summarized in Table 2. When assessing the Minkowski distance with p = 1 (Manhattan distance) and Euclidean distance with p = 2, we observed that the distances from the healthy voice data were shorter in order of benign mucosal diseases, vocal cord paralysis, and laryngeal cancer. Meanwhile, for measures representing similarity, such as cosine distance and correlation distance (with values ranging between 0 and 1, where values closer to 0 indicate higher similarity), the sequence of resemblance to healthy voice data is: benign mucosal diseases, followed by vocal cord paralysis, and then laryngeal cancer. This experiment represents the results of measuring the distance and similarity between MFCC-transformed signals, rather than the raw waveform of the audio data, to be used in the Convolutional Neural Network. From the perspective of healthy vocal signals, it is observed that there is a high degree of similarity between vocal cord paralysis and benign mucosal diseases signals when compared to healthy voice signals. This indicates that in binary classification tasks differentiating between healthy data and specific laryngeal disorders, distinguishing laryngeal cancer from healthy voice is a more challenging task than discriminating vocal cord paralysis, and distinguishing vocal cord paralysis is more challenging than distinguishing benign mucosal diseases.

**Table 2:** Similarity and distance of laryngeal cancer (cancer), vocal cord paralysis (paralysis) and benign mucosal disease (benign) vocalizations to healthy vocalizations.

Compared signals	Minkowski distance	Euclidean distance	Cosine distance	Correlation distance
	(p = 1)	(p = 2)		
Cancer and healthy	8968.71	455.29	0.60	0.23
Paralysis and healthy	8619.53	434.41	0.57	0.12
Benign and healthy	8004.90	406.75	0.49	0.06

Next, the an analysis of the similarity of laryngeal cancer to other benign conditions is shown in Table 3. “Regarding laryngeal cancer vocal signals, it can be observed that they exhibit a high degree of similarity with vocal cord paralysis and benign mucosal disease signals when compared to healthy vocal signals. It can be anticipated that in multi-class classification tasks, such as three-class or four-class classification involving not only distinguishing healthy from laryngeal cancer but also including other laryngeal disorders, the similarity in signal characteristics among classes increases, making the classification task more challenging.

**Table 3:** Similarity and distance of vocal cord paralysis and benign mucosal disease vocalizations to laryngeal cancer.

Comparison signals	Minkowski distance	Euclidean distance	Cosine distance	Correlation distance
	(p = 1)	(p = 2)		
Cancer and healthy	8968.71	455.29	0.60	0.23
Cancer and paralysis	8844.99	447.75	0.61	0.08
Cancer and benign	8510.98	433.24	0.52	0.07

### Binary classification: healthy vs Laryngeal cancer and other laryngeal diseases

First, we conducted experiments on binary classification tasks, where the goal was to classify healthy vocal signals against each individual vocal disorder.

#### Healthy vs laryngeal cancer

**Figure 2:**
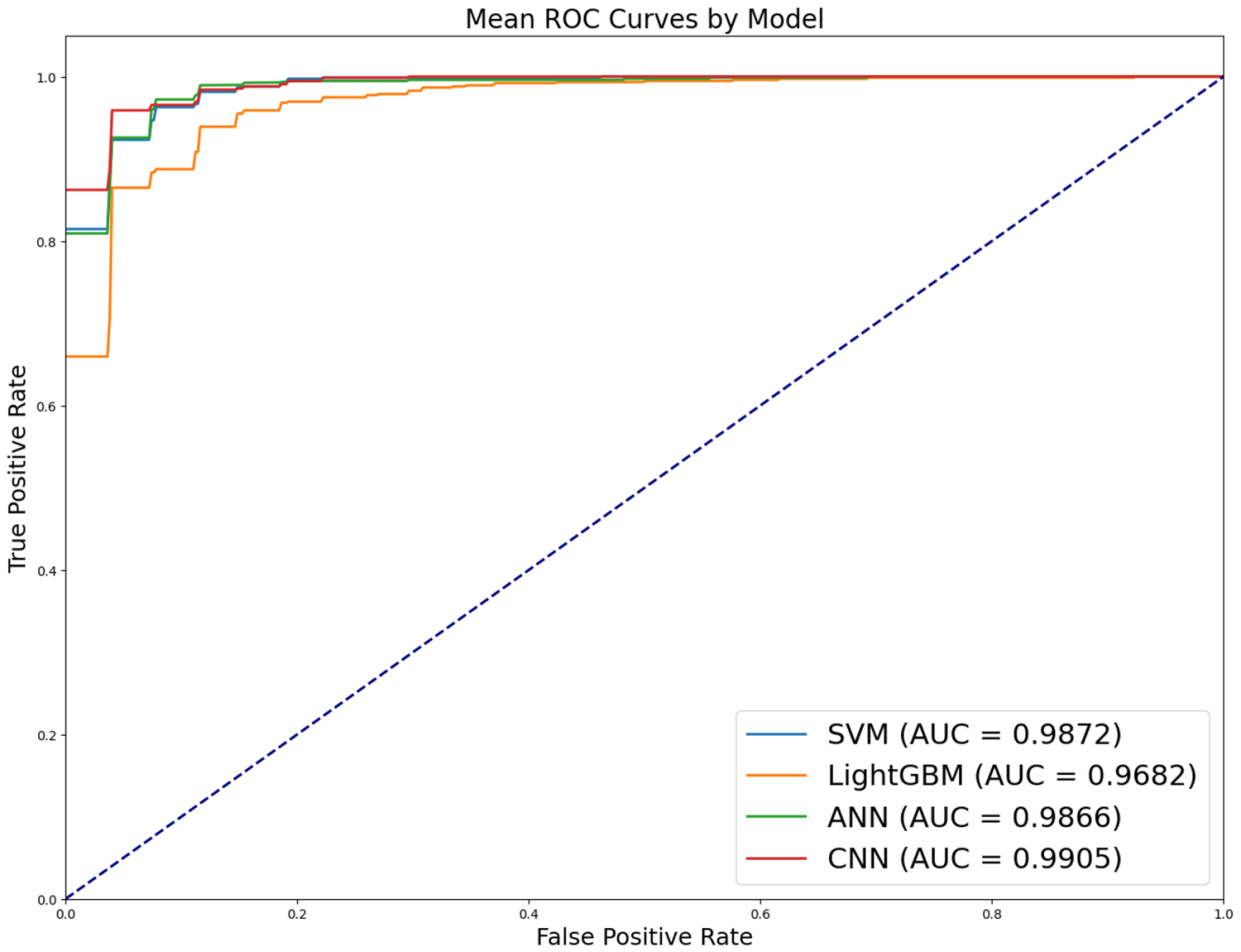
AUROC of classification between laryngeal cancer and healthy.

The result of the binary classification between healthy and laryngeal cancer vocalizations are shown in Table 4 and Fig. 2. ANN and CNN showed better performance than SVM and LightGBM in this task. The features of the MFCCs of healthy and laryngeal cancer speech are quite distinct in Table 2, so there is no performance improvement in applying the CNN after image conversion. These results demonstrate a significant improvement in accuracy by more than 0.25 compared to prior studies^12,13^. This improvement is attributed to the effect of segmenting the audio signals into fine-grained MFCC data, which enhances data augmentation while preserving key features. Additionally, the adoption of a deep-resnet architecture in the CNN model’s hidden layers led to these results. Moreover, the AUC performance exceeded that evaluated by experts for the same binary classification data, showing an improvement of 0.23 based on the AUC metric.

**Table 4:** Results of classification between laryngeal cancer and healthy (mean ± standard deviation of five-fold cross validation).

Model	Accuracy	Precision	Recall	F1-score
SVM	0.9425 ± 0.0170	0.8732 ± 0.0338	0.9322 ± 0.0086	0.8973 ± 0.0246
LightGBM	0.9302 ± 0.0116	0.8464 ± 0.0174	0.9158 ± 0.0308	0.8754 ± 0.0219
ANN	0.9651 ± 0.0156	**0.9578 ± 0.0297**	0.9024 ± 0.0400	0.9267 ± 0.0336
CNN	**0.9651 ± 0.0152**	0.9394 ± 0.0431	**0.9330 ± 0.0329**	**0.9326 ± 0.0258**

Maximum values are in [bold].

#### Healthy vs vocal cord paralysis

**Figure 3:**
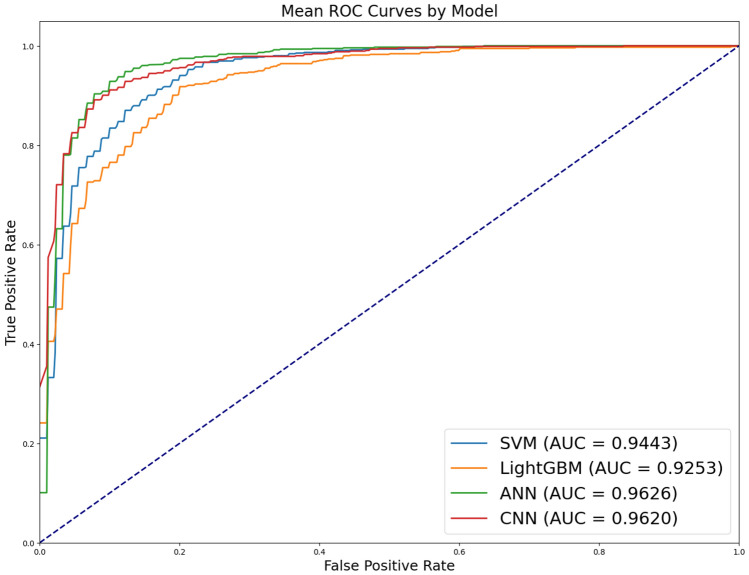
AUROC of classification between vocal cord paralysis and healthy.

The result of the binary classification between healthy and vocal cord paralysis vocalizations are shown in Table 5 and Fig. 3. As the difference between the voice MFCCs of healthy and vocal cord paralysis is smaller than that of healthy and laryngeal cancer cases as seen in Table 2, the accuracy of the classification is decreased. However, it should be noted that the performance of the ANN and CNN models exhibited a degradation compared to SVM and LightGBM, yet overall, the models still achieved a performance level exceeding 0.9. The signal similarity distance between vocal cord paralysis and healthy voice is closer than that with laryngeal cancer, indicating that vocal cord paralysis more closely resembles healthy voice signals. Consequently, this similarity results in lower classification performance of ANNs and CNNs for vocal cord paralysis compared to laryngeal cancer.

**Table 5:** Results of classification between laryngeal paralysis and healthy (mean ± standard deviation of five-fold cross validation).

Model	Accuracy	Precision	Recall	F1-score
SVM	0.8782 ± 0.0140	0.8714 ± 0.0137	0.8675 ± 0.0178	0.8692 ± 0.0158
LightGBM	0.8674 ± 0.0209	0.8597 ± 0.0232	0.8571 ± 0.0223	0.8581 ± 0.0222
ANN	0.9097 ± 0.0206	**0.9142 ± 0.0187**	0.8922 ± 0.0258	0.9009 ± 0.0235
CNN	**0.9105 ± 0.0204**	0.9101 ± 0.0227	**0.8991 ± 0.0219**	**0.9033 ± 0.0219**

Maximum values are in [bold].

#### Healthy vs benign mucosal disease

**Figure 4:**
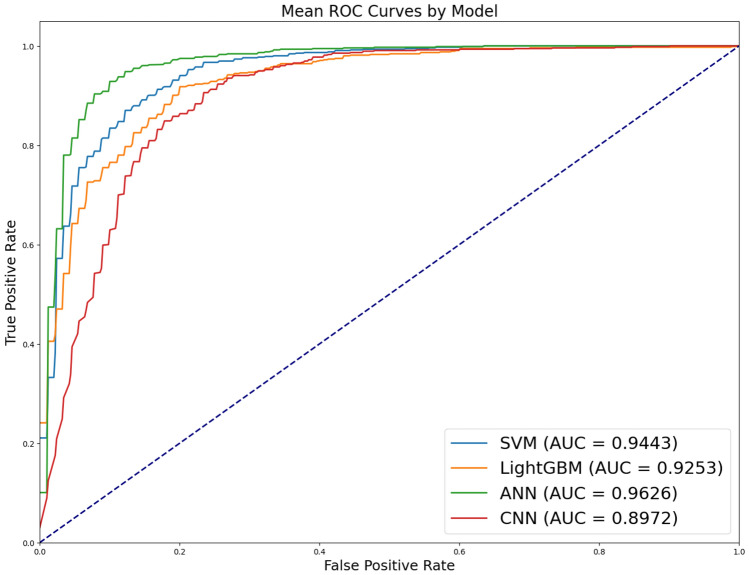
AUROC of classification between benign mucosal disease and healthy.

The result of the binary classification between healthy and vocal cord paralysis vocalizations are shown in Table 6 and Fig. 4. For benign mucosal diseases, the classification accuracy tends to decrease as the similarity to healthy data increases compared to laryngeal cancer and vocal cord paralysis. In this task, the CNN exhibits superior performance. Moreover, when there’s an increase in voice signal similarity between standard data and the disease, the CNN excels over the ANN. This can be attributed to the extraction of MFCC features via the convolutional layers. By transforming the voice signal, inherently time series data, into MFCC and subsequently visualizing the converted MFCC, it becomes evident that the application of CNN enables the identification of laryngeal cancer characteristics within the MFCC. These characteristics, typically challenging to detect, are effectively captured owing to the robust feature extraction capabilities of CNN. This process underscores the potential of CNN in enhancing the detection and analysis of subtle yet critical features in voice signal data, particularly those associated with laryngeal cancer, through the utilization of MFCC.

**Table 6:** Results of classification between benign mucosal disease and healthy (mean ± standard deviation of five-fold cross validation).

Model	Accuracy	Precision	Recall	F1-score
SVM	0.8033 ± 0.0247	0.7870 ± 0.0265	0.7829 ± 0.0334	0.7839 ± 0.0294
LightGBM	0.8032 ± 0.0214	0.7864 ± 0.0238	0.7833 ± 0.0249	0.7845 ± 0.0241
ANN	0.8297 ± 0.0228	0.8414 ± 0.0244	0.7827 ± 0.0279	0.7991 ± 0.0286
CNN	**0.8475 ± 0.0352**	**0.8524 ± 0.0291**	**0.8129 ± 0.0511**	**0.8235 ± 0.0478**

Maximum values are in [bold].

### Multiclass classification: healthy, laryngeal cancer, and vocal cord paralysis

We also evaluated the classification accuracy of AI models on a dataset that includes not only healthy and laryngeal cancer data but also 1–2 additional laryngeal disorders. The performance deterioration in multi-class classification with 3–4 classes compared to binary classification can be attributed to the increased complexity and overlap among multiple classes, making it more challenging for the AI model to distinguish and classify each class accurately. Furthermore, as indicated by the results of signal analysis, the addition of extra classes is expected to render the extraction of distinctive feature characteristics among classes more challenging, resulting in a lower classification accuracy. However, this will serve as an experiment to assess whether the proposed deep learning models are proficient in feature extraction for classification and how robust they are in this context.

#### Classification of healthy, laryngeal cancer, and vocal cord paralysis

The classification task were performed involving healthy vocalizations, vocal cord paralysis vocalizations, and laryngeal cancer vocalizations, and the results are shown in Table 7. The classification outcomes from this task are not as high as those from binary classifications like laryngeal cancer vs. healthy and vocal cord paralysis vs. healthy. Notably, the CNN surpasses both SVM and LightGBM across all measures and exhibits better performance than ANN, barring a slight variation in accuracy.

**Table 7:** Results of classification among healthy condition, laryngeal cancer, and vocal cord paralysis (mean ± standard deviation of five-fold cross validation).

Model	Accuracy	Precision	Recall	F1-score
SVM	0.8090 ± 0.0269	0.7540 ± 0.0353	0.8161 ± 0.0270	0.7690 ± 0.0331
LightGBM	0.8231 ± 0.0158	0.7686 ± 0.0120	0.7625 ± 0.0225	0.7647 ± 0.0166
ANN	**0.8261 ± 0.0273**	0.7294 ± 0.1180	0.6886 ± 0.0758	0.7010 ± 0.0933
CNN	0.8254 ± 0.0312	**0.7779 ± 0.0544**	**0.7842 ± 0.0587**	**0.7768 ± 0.0525**

Maximum values are in [bold].

#### Classification of healthy, laryngeal cancer, and benign mucosal diseases

The classification task were performed involving healthy vocalizations, benign mucosalvocalizations, and laryngeal cancer vocalizations, and the results are shown in Table 8. For the given task, its performance is not as robust as when classifying between healthy and the combined categories of laryngeal cancer and vocal cord paralysis. The heightened similarity observed between healthy and benign mucosal disease, as well as between benign mucosal disease and laryngeal cancer in Tables 2 and 3, is likely contributing to the misclassifications. The performance gap between ANN (and other machine learning models) and CNN is especially evident in the binary classification of benign mucosal disease versus healthy.

**Table 8:** Results of classification among healthy, laryngeal cancer, and benign mucosal diseases (mean ± standard deviation of five-fold cross validation).

Model	Accuracy	Precision	Recall	F1-score
SVM	0.7529 ± 0.0152	0.6994 ± 0.0219	0.7530 ± 0.0216	0.7133 ± 0.0193
LightGBM	0.7521 ± 0.0215	0.6896 ± 0.0246	0.7029 ± 0.0061	0.6924 ± 0.0149
ANN	0.7353 ± 0.0192	0.7239 ± 0.0727	0.5597 ± 0.0453	0.5657 ± 0.0607
CNN	**0.8202 ± 0.0026**	**0.8087 ± 0.0228**	**0.7572 ± 0.0222**	**0.7751 ± 0.0153**

Maximum values are in [bold].

#### Classification of healthy, laryngeal cancer, vocal cord paralysis and benign mucosal disease

Moreover, experiments were conducted to classify healthy vocalizations, benign mucosal disease vocalizations, and laryngeal cancer vocalizations. (Table 9) The performance declines in classification models that incorporate multiple benign mucosal conditions. Nonetheless, the CNN surpasses other models in all performance measures.ResNet results pertaining to specific conditions, for the 7 individuals with bilateral laryngeal cancer, a recall value of 0.75 was observed. For the 17 individuals with anterior commissure involvement laryngeal cancer, the recall was 0.69. Additionally, for those with anterior commissure involvement in benign mucosal disease, a recall of 0.67 was measured.

**Table 9:** Results of classification among healthy, laryngeal cancer, vocal cord paralysis and benign mucosal diseases (mean ± standard deviation of five-fold cross validation).

Model	Accuracy	Precision	Recall	F1-score
SVM	0.6828 ± 0.0224	0.6451 ± 0.0299	0.6905 ± 0.0310	0.6511 ± 0.0317
LightGBM	0.7152 ± 0.0309	0.6790 ± 0.0317	0.6769 ± 0.0388	0.6761 ± 0.0351
ANN	0.6407 ± 0.0284	0.6577 ± 0.0607	0.5192 ± 0.0530	0.5159 ± 0.0555
CNN	**0.7530 ± 0.0544**	**0.7515 ± 0.0445**	**0.7264 ± 0.0676**	**0.7253 ± 0.0699**

Maximum values are in [bold].

## Discussion

- Analysis of laryngeal cancer detection in multi-class classification

**Figure 5:**
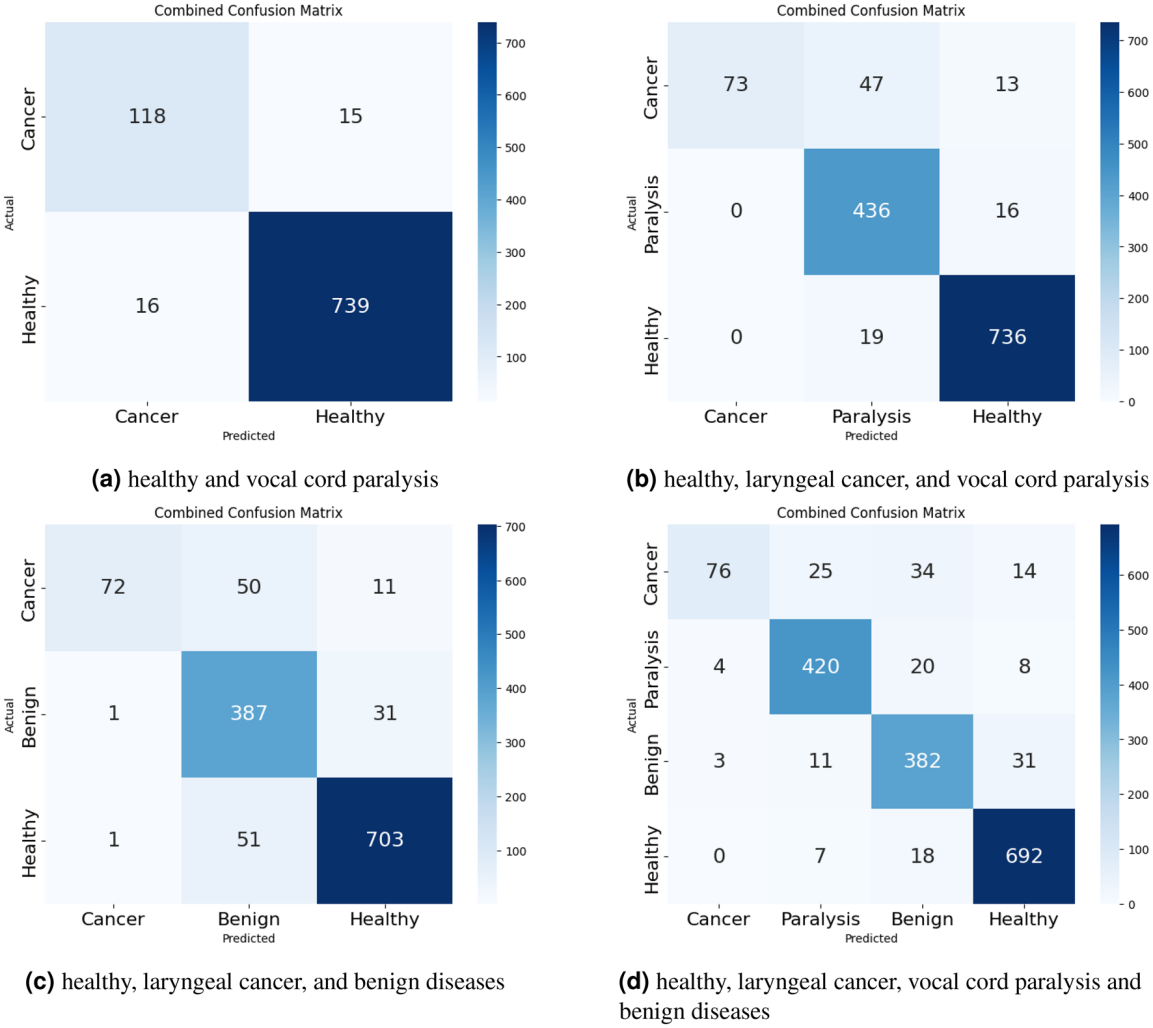
Confusion matrix of multi-class classification.

Significant advancements have been made in voice-based laryngeal cancer diagnosis. In binary classifications distinguishing between healthy voices and those with laryngeal cancer, accuracy rates have shown substantial improvement. For instance, Kim et al.^12^ reported an accuracy rate of 85$\%$. However, in our study, all models consistently achieved at least 93$\%$ accuracy. This remarkable improvement can be attributed to model development, an increased number of participants, and more precise participant classification. In the realm of multi-class classification, various approaches have been studied. For instance, Peng et al.^24^ categorized voices into four distinct classes: hyperkinetic dysphonia, hypokinetic dysphonia, flux laryngitis, and healthy, based on the SIFEL clinical standards proposed by the Italian Society of Phoniatrics and Logopaedics^25^. They achieved an impressive accuracy range of 97$\%$ to 99$\%$. While this classification method excels in distinguishing vocal fold movement, it may face challenges in distinguishing malignancy among voices. For this reason, there was no description about malignancy or laryngeal cancer. Hung et al.^15^ categorized voices into five classes: neoplasm, functional dysphonia, vocal palsy, phonotrauma, and healthy. The study did not provide a specific description of why it was divided into five categories. However, this system included neoplasm as one of the categories and reported an accuracy rate of 77.5$\%$. Hu et al.^14^ categorized voices into five classes: unilateral vocal paralysis, adductor spasmodic dysphonia, vocal atrophy, organic vocal fold lesions, and healthy voice. They also described no classification criteria for dividing it into five categories and reported 66.9 $\%$ of the accuracy. However, the types of voice disorders are very diverse, and the categorization systems for classifying voice disorders also vary among clinicians. Some voice disorders, although diagnosed differently, exhibit similar characteristics and treatment approaches, and thus can be classified under the same category. For example, vocal nodules and vocal polyps, despite their different mechanisms of development, can be classified as benign mucosal diseases because their treatments involve either voice therapy or microlaryngeal surgery. For these reasons, in contrast to previous studies, we have classified abnormal voices based on clinical treatment approaches and contact of the vocal cords, considering pathological results. Representative examples include laryngeal cancer, benign mucosal disease, vocal cord paralysis, and functional dysphonia. The treatment approaches for these diseases differ: laryngeal cancer must require radiotherapy or surgery, benign mucosal disease is managed with voice therapy or microlaryngeal surgery, vocal cord paralysis needs voice therapy or surgical approach including injection laryngoplasty, and functional dysphonia may be treated with voice therapy or botox injections. In this study, there were no patients with functional dysphonia because comprehensive sentence analysis is crucial to diagnose functional dysphonia such as spasmodic dysphonia and severe muscle tension dysphonia. With more data on sentence patterns and more development of AI technique, it may become possible for AI to detect functional dysphonia as well. In aspect of vocal cords contact, there are also notable distinctions among these diseases. Laryngeal cancer and benign mucosal disease usually exhibit stronger and rougher contacts between the vocal cords.^26–28^ The roughness of vocal cord contact is influenced not only by the size and location of the lesion but also by the hardness of the lesion. The hardness of the lesion varies depending on the contents, and the lesion of laryngeal cancer is generally harder than that of benign mucosal disease because malignancy lesion invades the basement membrane of vocal cord. In contrast, there was weaker or absent contact in vocal paralysis. Comprehensively, we prioritized the early detection of laryngeal cancer, focusing on clinical treatments that requires further treatment as soon as possible. To distinguish laryngeal cancer from other voice disorders in voice-based laryngeal cancer diagnosis, we considered the extent of vocal cord contact and whether a diagnosis could be determined through simple /a : / phonation. Consequently, we classified abnormal voices into laryngeal cancer, benign mucosal disease, and vocal cord paralysis. Because the ability to recognize laryngeal cancer is the most important performance indicator, we would like to analyze the laryngeal cancer detection rate based on the results of multi-class classification. For this purpose, we derived the average value of 5-fold cross validation of sensitivity and specificity values based on laryngeal cancer in multi-class classification. The confusion matrix for the individual multi-class classification validation results using CNN is shown in Fig. 5. When classifying laryngeal cancer, vocal cord paralysis, and healthy voices, the outcomes were: sensitivity at 0.7128, specificity at 0.9594, and AUC at 0.9347. For laryngeal cancer, benign disease, and healthy classification: sensitivity was 0.6162, specificity 0.9830, and AUC 0.9155. When all classes, including laryngeal cancer, vocal cord paralysis, benign disease, and healthy, were classified together, sensitivity stood at 0.6627, specificity at 0.9711, and AUC at 0.8742. Sensitivity is pivotal in medical diagnosis, and our results show a notable decline in sensitivity as benign diseases are incorporated. This limitation is attributed to the complexities arising from signal similarities and data imbalances. The current patient ratio across classes is 27:97:80:162 for Laryngeal cancer, vocal cord paralysis, benign mucusal disease, and healthy participants respectively. Post-MFCC voice data conversion, the distribution stands at 133:452:419:755 for the same categories. Enhancing model performance, especially sensitivity, may be achievable by addressing data imbalances, potentially via oversampling strategies^29,30^.

- t-SNE analysis of multi-label classification

**Figure 6:**
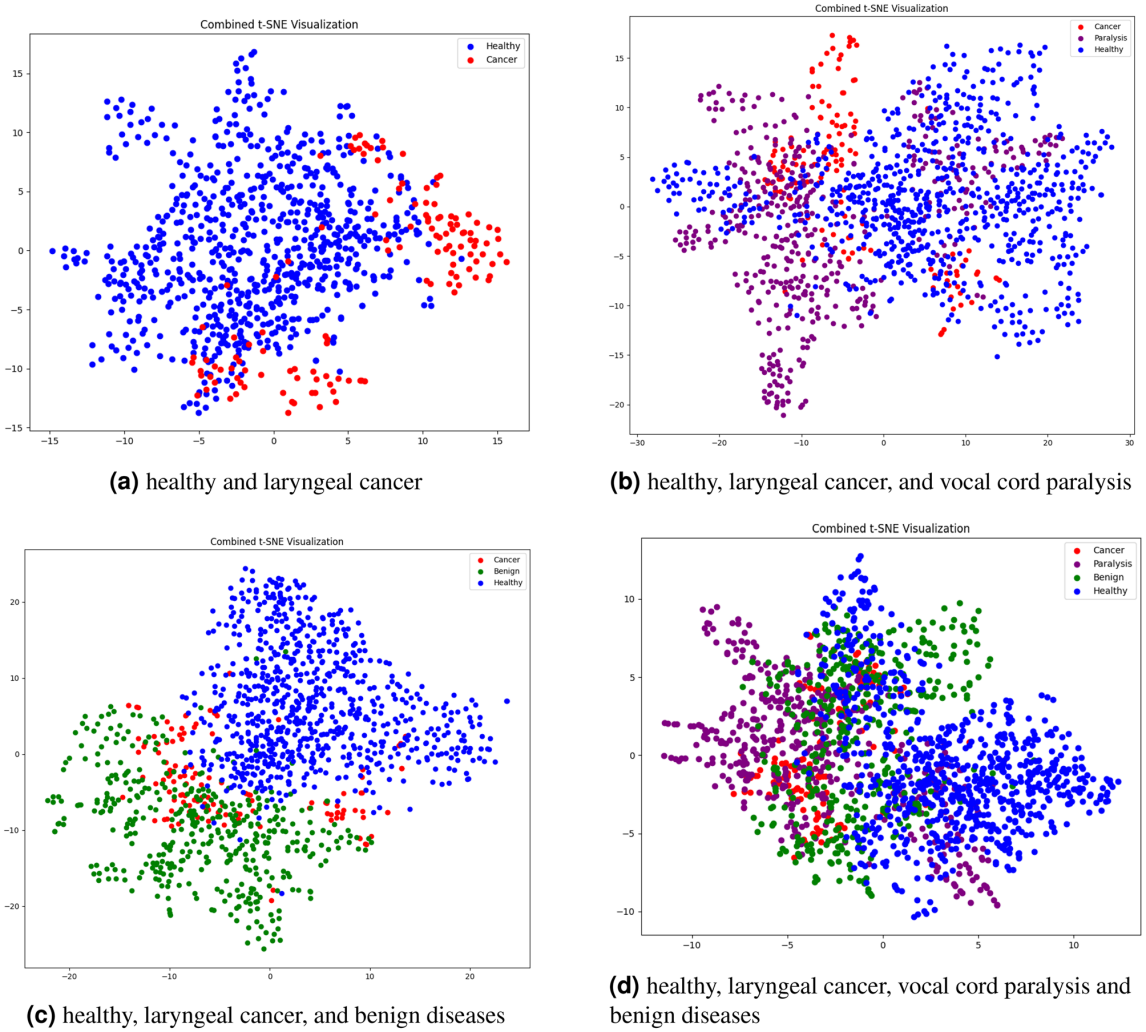
feature analysis using t-SNE of classification for laryngeal cancer.

To delve deeper into the performance decline of the classification model when multiple benign diseases are incorporated with laryngeal cancer, we examined the feature map of the CNN classifier tailored for the dataset inclusive of laryngeal cancer. We utilized t-Distributed Stochastic Neighbor Embedding (t-SNE)^31^, a dimensionality reduction technique, to visualize high-dimensional data by preserving local data structures within a lower-dimensional space. This method effectively reveals patterns or clusters by converting data similarities into joint probabilities and minimizing their divergence between high and low dimensions, making t-SNE a popular tool for exploratory data analysis. Using t-SNE, we processed the CNN model’s feature maps by pooling global averages. As illustrated in Fig. 6, the introduction of benign diseases leads to closer or overlapping features between classes, which in turn compromises the classification accuracy.

- Limitations

There are several limitations in this study. First, this study was done by only the voice of men. As voice is affected by sex significantly, there had to be differences in study direction and methods according to sex. Furthermore, the types of laryngeal disease also vary depending on sex. In Korea, approximately 90$\%$ of laryngeal cancer cases occur in men^3^, and in the United States, approximately 84$\%$ of cases occur in men^32^. In contrast, the occurrence of benign mucosal diseases has very different sex ratios across studies. Some studies report a male-to-female ratio of 2:1^33–35^, but others report a higher female-to-male ratio^36^ For these reasons, analysis of women’s voices will be conducted in different method from that of men. Second, the number of the laryngeal cancer patients is insufficient. The occurrence rate of the laryngeal cancer in the United States was 2.26 per 100,000 people in 2018^37^. In Korea, about 1200 people were diagnosed as laryngeal cancer in 2020. For these reasons, the number of laryngeal cancer cases in our study is smaller compared to other laryngeal diseases. Increasing the enrollment of laryngeal cancer patients would likely yield more accurate results. Third, our classification criteria require further elaborations. Notably, functional dysphonia is not included in our study due to its complex diagnostic nature, for which we believe sentence analysis is crucial. Additionally, the number of functional dysphonia cases in our study was limited. We included sulcus vocalis without voice discomfort in the healthy group, as it typically does not require treatment, while we excluded cases with sulcus vocalis with voice discomfort. Similarly, granuloma cases discovered incidentally were excluded, whereas those with voice discomfort were included as benign mucosal disease based on the principle that clinical symptoms warranted inclusion. In this study, supraglottic cancer case was not involved, because laryngeal cancer limited only on supraglottis affects rarely to voice. However, transglottic cancer cases were included. Phonetically, supraglottic cancer is thought to only affect the vocal tract, similar to oropharynx cancer such as tongue base cancer, and this should be studied in more detail in follow-up studies. Fourth, all data in this study were obtained in single center, and our study needs validation by other institution.

## Conclusion

The study focuses on classifying laryngeal cancer, vocal cord paralysis, benign mucosal diseases, and healthy cases by transforming speech into MFCCs and employing various classification approaches. Utilizing our proposed parameters for MFCC transformation and analyzing the transformed MFCCs through ResNet for diagnosing laryngeal cancer showed the highest detection rates in both binary and multi-class classifications. These findings indicate a slight improvement in performance over previously published papers, demonstrating the efficacy of our methodology in enhancing the accuracy of laryngeal cancer detection. Building on this findings, we plan to develop a more effective model for screening laryngeal cancer from sentence-level voice files containing a variety of phonemes, not limited to the /a/ phoneme. Additionally, we aim to address the data imbalance issue prevalent in laryngeal cancer datasets through data augmentation methods using generative models, potentially improving the robustness and performance of our diagnostic model.
